# Enhancing the Efficiency of Distraction Osteogenesis through Rate-Varying Distraction: A Computational Study

**DOI:** 10.3390/ijms222111734

**Published:** 2021-10-29

**Authors:** Ruisen Fu, Yili Feng, David Bertrand, Tianming Du, Youjun Liu, Bettina M. Willie, Haisheng Yang

**Affiliations:** 1 Department of Biomedical Engineering, Faculty of Environment and Life, Beijing University of Technology, Beijing 100124, China; furuisen@emails.bjut.edu.cn (R.F.); fengyili0@163.com (Y.F.); dutianming@bjut.edu.cn (T.D.); lyjlma@bjut.edu.cn (Y.L.); 2 Department of Pediatric Surgery, McGill University, Montreal, QC H4A 3J1, Canada; david.bertrand@mail.mcgill.ca (D.B.); BWillie@shriners.mcgill.ca (B.M.W.); 3 Research Centre, Shriners Hospital for Children-Canada, Montreal, QC H4A 0A9, Canada

**Keywords:** distraction osteogenesis, rate-varying distraction, finite element analysis, mechano-regulatory tissue differentiation, bone regeneration

## Abstract

Distraction osteogenesis (DO) is a mechanobiological process of producing new bone and overlying soft tissues through the gradual and controlled distraction of surgically separated bone segments. The process of bone regeneration during DO is largely affected by distraction parameters. In the present study, a distraction strategy with varying distraction rates (i.e., “rate-varying distraction”) is proposed, with the aim of shortening the distraction time and improving the efficiency of DO. We hypothesized that faster and better healing can be achieved with rate-varying distractions, as compared with constant-rate distractions. A computational model incorporating the viscoelastic behaviors of the callus tissues and the mechano-regulatory tissue differentiation laws was developed and validated to predict the bone regeneration process during DO. The effect of rate-varying distraction on the healing outcomes (bony bridging time and bone formation) was examined. Compared to the constant low-rate distraction, a low-to-high rate-varying distraction provided a favorable mechanical environment for angiogenesis and bone tissue differentiation, throughout the distraction and consolidation phase, leading to an improved healing outcome with a shortened healing time. These results suggest that a rate-varying clinical strategy could reduce the overall treatment time of DO and decrease the risk of complications related to the external fixator.

## 1. Introduction

Repair of large bone defects remains a challenge in orthopaedics. Although numerous techniques are available as treatment options, including autogenous bone grafts, allografts, bone graft substitutes, and vascularized fibular bone grafts [1,2,3], distraction osteogenesis (DO)—an in vivo tissue engineering approach—remains the clinical gold standard for treating large bone defects [4,5].

DO is a mechanobiological process which involves producing new bone and overlying soft tissues through the gradual and controlled distraction of surgically separated bone segments [6,7,8]. DO consists of three consecutive phases: latency, distraction, and consolidation phases [6,9]. The process of bone regeneration during DO is regulated by mechanical stimuli; therefore, the healing outcomes are largely affected by distraction parameters such as the distraction rate and frequency [6,9,10,11]. One important mechanical parameter is the distraction rate (i.e., the distance over which the bone is lengthened per day) [10,11]. Several preclinical studies have demonstrated that extremely high distraction rates (2.7 mm/day) disrupt angiogenesis and inhibit bone formation [12,13]; whereas, a very low rate (0.3 mm/day) does not maximally stimulate angiogenesis, resulting in premature bone formation [12,13]. Ilizarov previously proposed an appropriate distraction rate to be 1 mm/day for DO [10,11], which was supported by other animal studies [12,13] and has been commonly used in clinic practices [8,14]. However, for clinical cases where lengthening exceeds 5 cm, distractions of 1 mm/day are associated with a prolonged treatment period, leading to various complications and increased numbers of revision surgeries [14,15]. Therefore, there remains a critical need to optimize the distraction parameters to shorten the distraction time and improve the efficiency of DO [6].

To shorten the distraction period for DO, one possible strategy reported by Long et al. [16] and Schiller et al. [17] was to administer growth factors at an appropriate timing when high distraction rates were applied. This may compensate for delayed callus formation caused by a high distraction rate [16]. However, due to a variety of reasons including inconsistent results and ectopic bone formation, the use of growth factors has largely fallen out of favor [16,17,18]. Another strategy that is safer, less costly, and easier to operate, is to manipulate the distraction parameters, for example combining high-frequency distractions with high-rate distractions [19]. However, too many distractions per day may increase the incidence of complications [19], and therefore may not be clinically applicable. A relatively low rate of distraction benefits angiogenesis, but a high rate allows for a shortened distraction period. A potential solution would be a distraction strategy with varying distraction rates (i.e., “rate-varying distraction”) to reduce the duration of the distraction period, while maintaining an optimal mechanical environment for angiogenesis and bone formation. To our knowledge, no studies have explored the efficacy of any rate-varying distraction protocols. Questions remain concerning the issue of how to vary the distraction rate to achieve fast and optimal healing, and surrounding what its underlying mechano-regulatory mechanisms are.

An in silico simulation is well-suited to address this issue because “unlimited” numbers of protocols, with various combinations of distraction rates, can be designed and tested. Finite element modeling somewhat overcomes the limitations of in vivo animal or clinical studies where only limited protocols can be examined and high variability is often observed between subjects. Hence, the aim of the present study was to comprehensively examine the effect of rate-varying distractions on healing outcomes, using a computational DO model developed and validated using previously published experimental data in sheep [4]. We hypothesized that faster and better healing can be achieved with rate-varying distractions, compared to constant-rate distractions.

## 2. Results

### 2.1. Computational Predictions and Experimental Validation of the Bone Regeneration Process under the Constant Low-Rate Distraction Protocol

**Figure 1:**
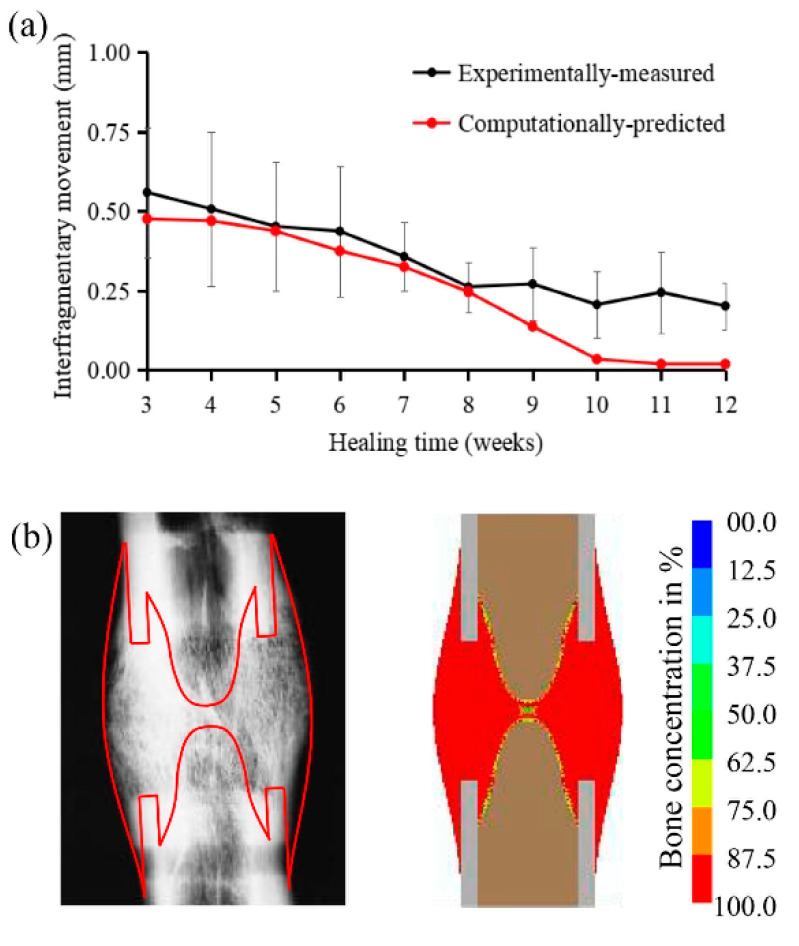
(**a**) Comparisons between computationally predicted interfragmentary movements and experimentally measured interfragmentary movements (mean ± SD), as a function of the healing time, in a bony defect model of sheep metatarsus [4]. (**b**) Left: radiograph of an osteotomized sheep metatarsus, 12 weeks postoperatively [4]. Right: our model-predicted bone tissue concentrations after 12 weeks.

Under the constant low-rate distraction protocol (1mm/day, twice a day for 15 days) our model-predicted interfragmentary movements (IFMs) of the distraction gap were compared against those measured from the in vivo experimental data [4] (Figure 1a). The change in IFMs, as a function of the healing time, showed a good consistency between predicted and measured values (Figure 1a). Generally, the IFMs decreased as healing progressed, reaching a stable value of around 0.02 mm, which was indicative of a complete bony union [20]. The healed bone tissue distributions were also consistent between radiographic observations and model predictions (Figure 1b).

**Figure 2:**
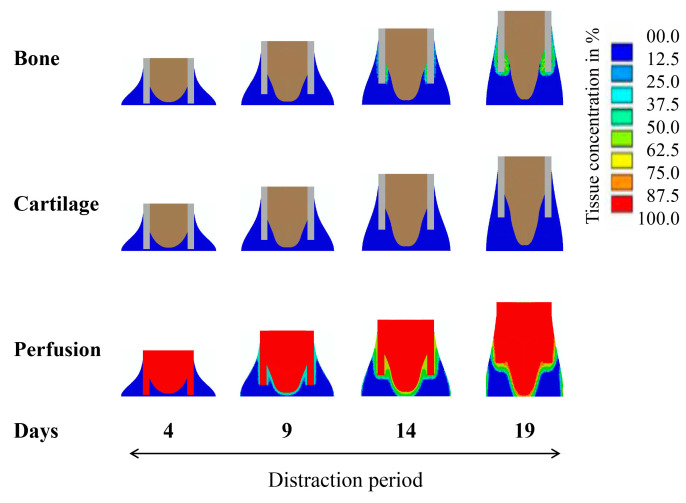
Model-predicted concentrations of bone, cartilage, and blood perfusion over the course of distraction (days 4–19) under the standard constant-rate protocol. Distraction rate and frequency were identical to the experimental setting, i.e., 1 mm/day, twice a day.

**Figure 3:**
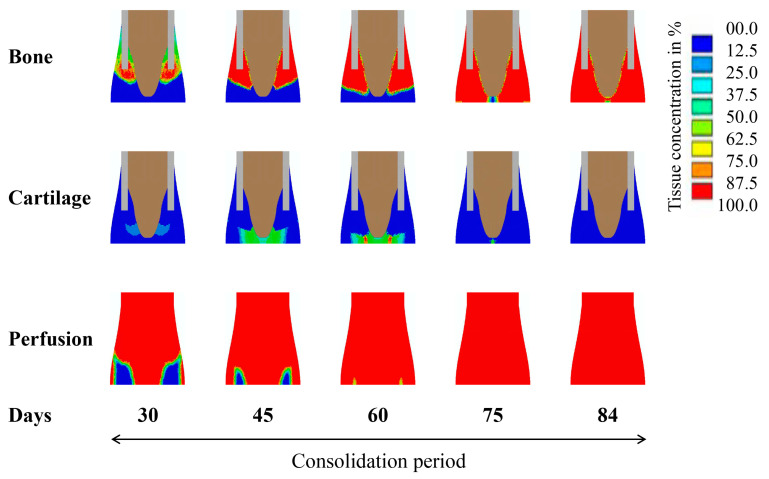
Model-predicted concentrations of bone, cartilage, and blood perfusion over the course of consolidation (day 19–84) under the standard constant-rate protocol. Distraction rate and frequency were identical to the experimental setting, i.e., 1 mm/day, twice a day.

According to the model predictions, we observed that the blood perfusion in the callus showed a slow recovery in the initial stage of distraction (days 4–9) (Figure 2). On day 14, with the recovery of blood perfusion in the callus area, new bone formed around the edge of the cortical bone. At the end of the distraction period (day 19), the blood perfusion around the cortical edge was fully restored and an appreciable amount of bone was regenerated through intramembranous ossification in the areas with high blood perfusion (Figure 2). During the consolidation period, the blood perfusion in the callus was gradually recovered and completed, while new bone grew along the direction of distraction (Figure 3). Cartilage was formed and then gradually replaced by bone tissue through cartilage calcification (Figure 3). At week 12 (day 84), the callus area was completely filled with newly formed bone tissues.

### 2.2. Influence of “Rate-Varying” Distractions on Bone Healing

**Figure 4:**
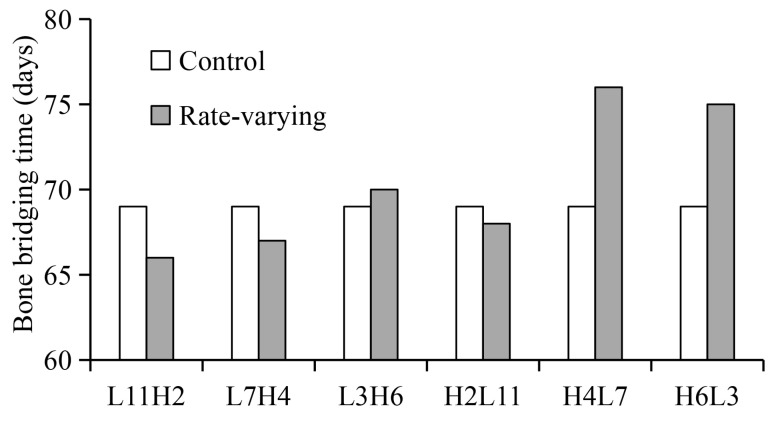
Bony bridging times for the constant-rate distraction protocol (control) and several typical rate-varying distraction protocols (L11H2, L7H4, L3H6, H2L11, H4L7, and H6L3). L11H2 indicates low-rate (L) distractions for 11 days followed by high-rate (H) distractions for 2 days. Control represents the standard constant-rate distraction protocol (1 mm/day for 15 days).

**Figure 5:**
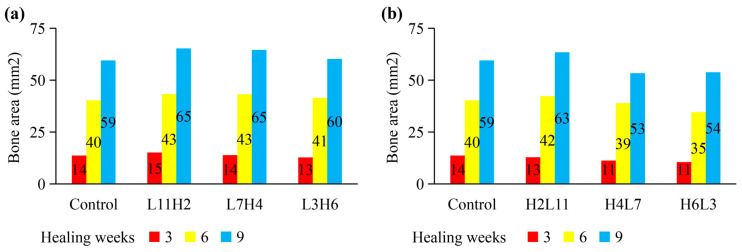
Newly generated bone area (mm^2^) within the callus at weeks 3, 6, and 9, respectively, under the low-constant-rate distraction protocol (control) as well as under various low-to-high rate (**a**) or high-to-low rate (**b**) protocols. L11H2 indicates low-rate (L) distractions for 11 days followed by high-rate (H) distractions for 2 days. Control represents the standard constant-rate distraction protocol (1 mm/day for 15 days).

Compared to the constant low-rate distraction, the rate-varying distraction protocols—with a low rate being followed by a high rate (e.g., L11H2 and L7H4)—generally induced better healing outcomes, including reduced time to bony bridging (Figure 4), reduced total healing time, and enhanced bone formation (Figure 5a). Contrary to these findings, high-to-low rate-varying distractions (e.g., H4L7 and H6L3) delayed bone healing and reduced bone formation, relative to the constant low-rate distraction (Figure 4 and Figure 5b).

**Figure 6:**
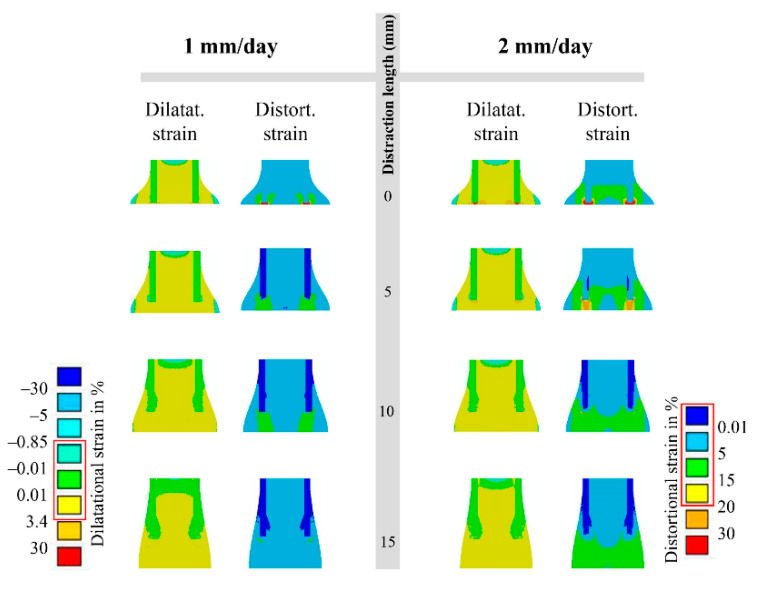
Dilatational (ε) and distortional (γ) strain states generated within the callus when the gap was lengthened by 0, 5, 10, and 15 mm under a constant low rate of 1 mm/day (left), or under a constant high rate of 2 mm/day (right). The red box corresponds to mechanical stimulation thresholds for bone formation in a mechano-regulatory tissue differentiation model (Figure 10).

In terms of the mechanical environment produced by the low-rate distraction of 1 mm/day, the interfragmentary strain (IFS) level was high (ε > 3.4 and γ > 30) at the very beginning of the distraction phase (distraction length 0–5 mm), was reduced to a moderate level (0.01 < ε < 3.4 and γ < 15) at the middle stage (distraction length 5–15 mm) that lasted for a relatively long term, and was reduced to a very low level (0.01 < ε < 3.4 and γ < 5) at the end of the distraction phase (distraction length 15 mm) (Figure 6). Under the high-rate distraction of 2 mm/day, high levels of IFSs (ε > 3.4 and γ > 20) were generated early and lasted for a relatively long period (e.g., distraction length 0–10 mm), reducing to a moderate level (0.01 < ε < 3.4 and γ < 15) afterwards (Figure 6).

**Figure 7:**
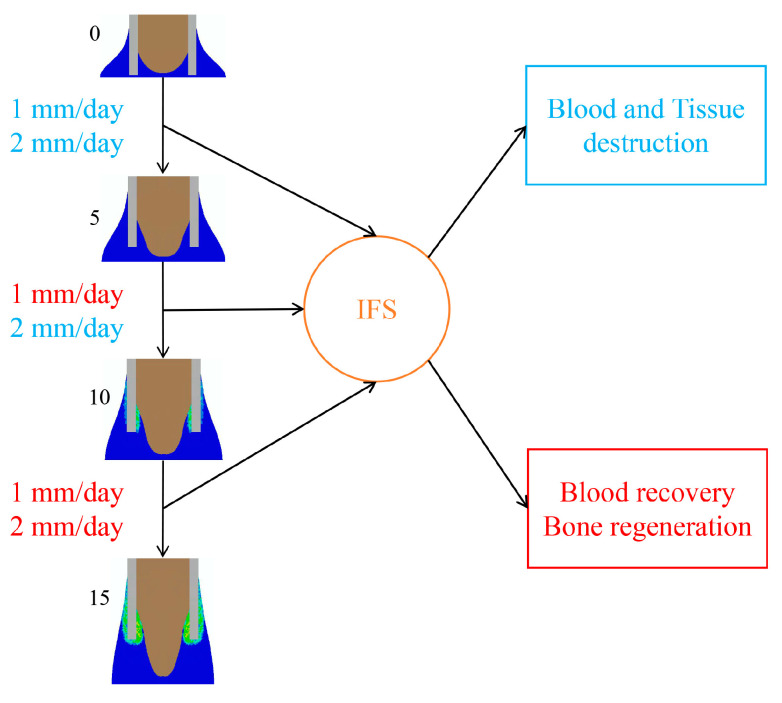
When the gap was lengthened by 0, 5, 10, and 15 mm under a constant low rate of 1 mm/day, or under a constant high rate of 2 mm/day, the interfragmentary strain (IFS) generated by the different distraction rates in the callus resulted in suitable (red) or disruptive (blue) blood recovery and bone regeneration.

**Figure 8:**
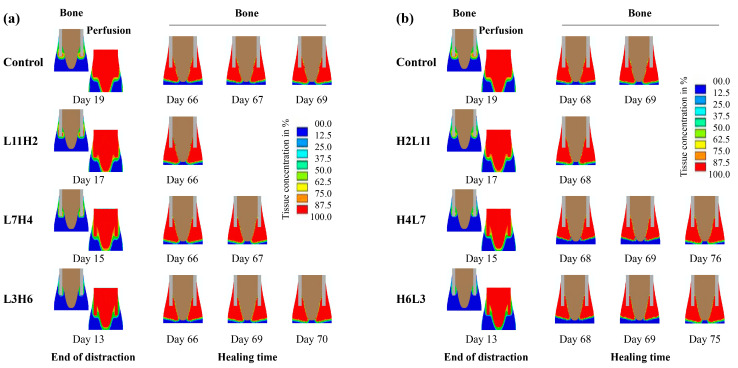
Distributions and concentrations of bone and blood perfusion at the end of the distraction phase, as well as at respective bony bridging times for the constant-rate distraction protocol (control) and several typical (**a**) low-to-high (L11H2, L7H4 and L3H6) or (**b**) high-to-low (H2L11, H4L7 and H6L3) rate-varying distraction protocols. L11H2 indicates low-rate (L) distractions for 11 days followed by high-rate (H) distractions for 2 days. Control represents the standard constant-rate distraction protocol (1 mm/day for 15 days).

When the low-rate distraction was first applied (e.g., L11—distraction length of 11 mm), the moderate IFS levels (0.01 < ε < 3.4 and γ < 15) (Figure 6) provided favorable mechanical environments for blood perfusion and bone formation (Figure 7). Since a high-rate distraction at a later stage of the distraction phase (distraction length of over 10 mm: Figure 6) also produced a moderate IFS environment (0.01 < ε < 3.4 and γ < 15), a combination of early low-rate distractions with a late high-rate distraction enhanced angiogenesis and bone regeneration (e.g., L11H2: Figure 4, Figure 5a and Figure 8a). However, when the high-rate distraction was applied at early stages of the distraction phase (e.g., L3H6 and H4L7), the induced high IFSs (ε > 3.4 and γ > 20) were outside of the mechanical window governing bone tissue differentiation (Figure 6 and Figure 7) and caused tissue damage and inhibited angiogenesis, leading to a reduction in bone formation and blood perfusion at the end of the distraction phase (Figure 7 and Figure 8). Eventually, delayed healing throughout the entire consolidation phase was observed (Figure 4 and Figure 8).

## 3. Discussion

A computational model, incorporating the viscoelastic behaviors of the callus tissues and the mechano-regulatory tissue differentiation laws, was developed and validated to predict the bone regeneration process during DO. The effect of rate-varying distractions on healing outcomes (bony bridging time and bone formation) was examined. Our results showed that, compared with the constant low-rate distraction, a low-to-high rate-varying distraction (e.g., L11H2 or L7H4) provides a favorable mechanical environment for angiogenesis and bone tissue differentiation throughout the entire distraction and during the subsequent consolidation phase—leading to an improved healing outcome with a shortened treatment time. These results suggest that a low-to-high rate-varying distraction can provide significantly better outcomes compared to constant-rate distraction. More importantly, rate-varying distractions offer an important strategy to manipulate the mechanical environment within the distraction gap to enhance angiogenesis and osteogenesis, ultimately promoting bone regeneration and reducing the length of the post-distraction consolidation period. 

The positive or negative healing results produced by different rate-varying distraction protocols can be explained by the mechanical environments induced within the distraction gap (Figure 6 and Figure 7). The strain environment within the gap is closely related to the gap size and the distance lengthened during each distraction (Figure 6). Under a constant-rate distraction, the IFS level continues to decrease due to the increasing gap size. For the low-rate distraction protocol (1 mm/day or 0.5 mm/action) used in the study, the IFSs (0.01 < ε < 3.4 and γ < 15) that are favorable for bone tissue differentiation occur after an early stage of the distraction phase (distraction length from 5 to 15 mm). For the high-rate distraction (2 mm/day or 1 mm/action), this bone-favorable strain environment comes later (distraction length from 10 to 15 mm). Therefore, a combination of these two optimal mechanical environment-associated distraction rates (e.g., L11H2) would achieve positive healing outcomes, relative to constant-rate distractions. A high-to-low distraction rate (e.g., H4L7 or H6L3) induces a detrimental healing outcome because this protocol results in a high IFS environment (ε > 3.4 and γ > 20) that exists for too long in the initial stage of distraction, which is not favorable for blood recovery and bone formation. High levels of IFS during the early phase have also been shown, both computationally and experimentally, to be deleterious in other healing scenarios for similar reasons [21,22]. However, it should be noted that H2L11 gives a comparable healing outcome to the constant low-rate distraction. This is because the strain level is high at the very early stages of the low-rate distraction (ε > 3.4 and γ > 30); therefore, replacing it with an even higher strain would not have a significant detrimental effect on healing. This would in turn suggest that a “very low–low–high” rate-varying distraction may be even more effective.

In addition to the useful findings provided by this study, the current computational model may offer a valuable tool for the clinical design of effective rate-varying distraction protocols. However, this type of computational simulation is technically complex and time-consuming. Alternatively, it might be more useful to propose an intuitive equation, considering the mechano-regulatory mechanisms above (Equation (1)). We could assume that the effective strain *ε*_eff_ is the primary mechanical stimulus regulating bone regeneration. The effective strain can be estimated based on the gap size or length (*l*) and the distraction rate (*r*):

*ε*_eff_ = *r*/*l* (1)

Since the optimal effective strain and the targeted distraction length are known, we could calculate the desired distraction rate for the different stages of the distraction phase, and subsequently design the most effective rate-varying distraction protocols to achieve optimal mechanical environments throughout the entire distraction phase. This equation may be very useful for a preliminary design of effective rate-varying distraction protocols. 

This is the first study investigating a novel rate-varying distraction concept. Although our findings regarding the effects of rate-varying distractions on DO may still require further validation from animal studies or clinical studies, our model-predicted healing results for the constant low-rate distraction are in line with previous constant-rate distraction experimental and computational observations [12,13,23]. Previous studies have demonstrated that a higher rate of distraction leads to the destruction of callus tissues and blood vessels, causing a delay in bone healing [12,13], while a relatively low-rate distraction is favorable for angiogenesis and osteogenesis, and can therefore promote bone regeneration [12,13].

One novel aspect of our computational model is that we took the time-dependent viscoelastic behavior of the callus tissues into account, based on experimental measurement. The existing numerical models of DO often regard callus tissues as a poroelastic material [23,24,25]. However, the constitutive parameters used in these models are not always based on experimental models of DO. Previous experimental studies have demonstrated a strong stress–relaxation phenomenon of the callus tissues following each step of distraction. Based on those experimental measures [26], we established a viscoelastic material model and used it in our computational models. The model’s predictions of the constant low-rate distraction protocol used in this study are in an excellent agreement with the experimental results (Figure 1).

Our study has several potential limitations. Firstly, to our knowledge, this is the first study comprehensively examining a rate-varying distraction concept. Despite the insights provided by the in silico simulation, future experimental studies are warranted to confirm our findings. Secondly, the exact definition of the low- or high-rate distractions might slightly affect our results. However, the primary take-home message would not change, based on the mechanism that we explained earlier in the discussion. We defined our low-rate distraction to be 1mm/day which is widely used and has been shown to be an optimal protocol. The aim of our study was to explore if any rate-varying distraction protocols could provide faster and improved healing outcomes.

In summary, our results demonstrate that a low-to-high rate-varying distraction strategy can provide faster and improved DO outcomes compared to a constant-rate distraction. A rate-varying strategy could reduce the overall treatment time of DO and thereby improve a patient’s quality of life, decreasing the risk of complications associated with the external fixator. 

## 4. Materials and Methods

### 4.1. Finite Element Modeling of the Distraction Site

**Figure 9:**
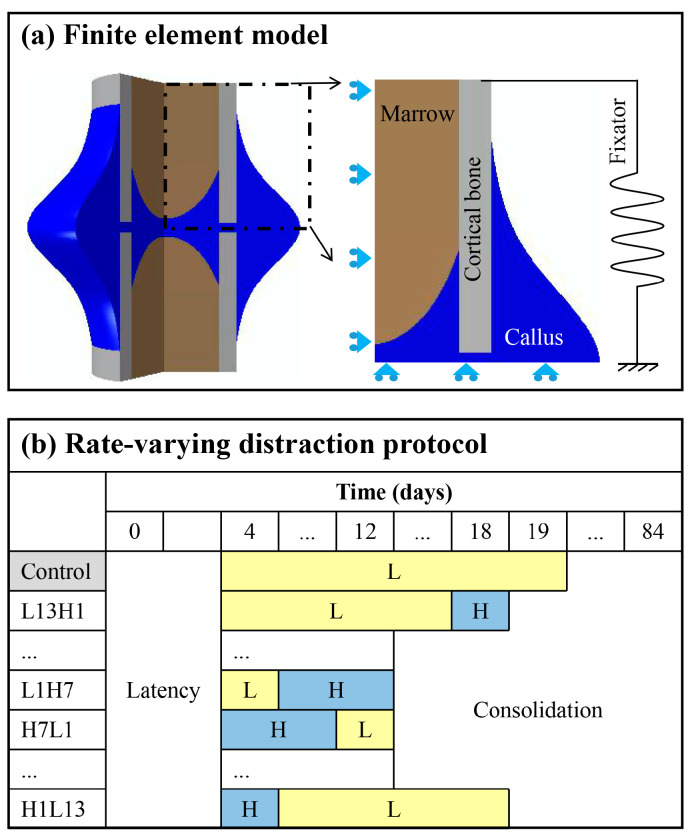
(**a**) A two-dimensional axisymmetric finite element model of the osteotomized site was created according to experimental measurements of the sheep metatarsus geometry [27]. The initial osteotomy gap was 1 mm. The inner and outer diameters of the cortical bone are 12 mm and 16 mm, respectively. The fixator was modeled as a nonlinear spring. (**b**) Different rate-varying distraction protocols were designed while the total distraction length of 15 mm was maintained. L indicates a low distraction rate (1 mm/day). H indicates a high distraction rate (2 mm/day). H1L13 indicates a high-rate distraction for 1 day, followed by low-rate distractions for 13 days. Control represents the standard constant-rate distraction protocol (1 mm/day for 15 days).

A two-dimensional axisymmetric finite element model of an osteotomized cortical bone was created (endosteal diameter: 12 mm, periosteal diameter: 16 mm, gap size: 1 mm) (Figure 9a). The geometry dimensions of the model were derived from a previous experimental study on sheep metatarsus [27]. In the finite element model, the fixator was represented with a nonlinear spring fixed at the bottom surface, rigidly connected to the top surface of the cortical bone fragments. The modeled fixator system was a customized external fixator used in the experiment [4]. The fixator was only considered during the consolidation phase, where physical activities play important roles—via the fixator—in influencing the mechanical environment within the distraction gap. In the distraction phase, distraction dominated the mechanical environment. A detailed description of the loading and boundary conditions is shown in (Figure 9a).

It has been experimentally shown that callus tissues exhibit time-dependent viscoelastic behavior under distraction loads [26,28,29]. After a distraction load was applied, the stresses within the distraction gap relaxed at an exponential rate. To reflect this phenomenon, we assigned viscoelastic material properties to the callus tissues. The linear elastic parts of the material properties (connective tissue, cartilage, woven bone, and cortical bone) were taken from Niemeyer et al. [30]; however, the exact constitutive parameters required to describe the viscoelastic behavior were largely unknown. Therefore, we fitted the daily relaxation curves (Equations (2) and (3)), obtained from the DO experiments in vivo [26], using a two-term Prony series in ABAQUS (ABAQUS, v 6.14-1, Dessault Systèmes Simulia Corp).

(2) $$F_{j}\left(t\right)=u(3.57e^{0.17j+\frac{-t}{3690}}+6.79e^{0.11j+\frac{-t}{469.3}}+6.89e^{0.08j+\frac{-t}{9.95e^{0.09j}}}+1.22e^{0.13j})$$

(3) $$F_{\mathrm{res}.j}=6.23{\%F}_{j}\left(0\right)$$

where *F* is the traction force, *t* is the time in seconds after the displacement increment u is applied, *j* represents the distraction day, *F*_res_ is a maximum residual force value through the callus after each step of distraction, and *F*_j_(0) is the peak traction force value on a given distraction day (*j*). In the initial state, the viscoelastic Prony coefficients of the callus tissues were g_11_ = 0.396, g_22_ = 0.542, τ_11_ = 10.775, and τ_22_ = 977.88, where g_11_ and g_22_ were weighting factors, and τ_11_ and τ_22_ were the time constants in the two-term Prony series.

During the distraction phase, the mechanical stimulation consisted of an applied displacement on the top of the cortical bone. The displacement was applied for 1 s and the final position was maintained for 12 h (relaxation period). During the consolidation phase, an axial compressive load of 500 N was applied to the model. This load represents the primary physiological axial loading generating on metatarsus of the sheep during normal walking [27].

### 4.2. Mechanobiological Simulation of Distraction Osteogenesis

**Figure 10:**
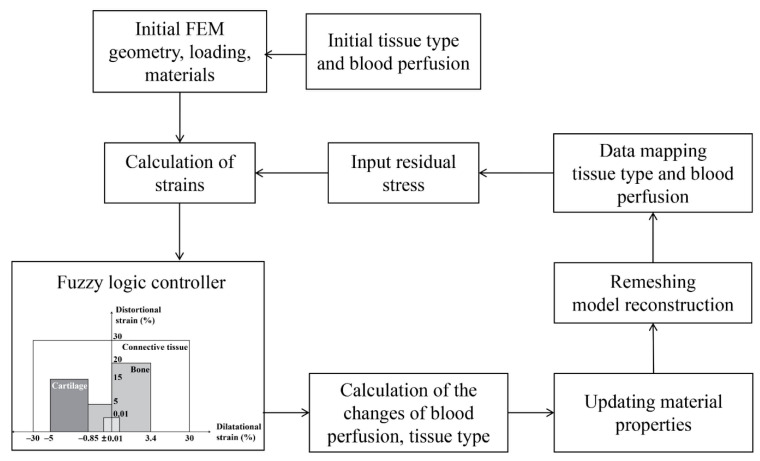
Flow chart for the numerical implementation of the mechanobiological simulation of the bone regeneration process during DO, including the tissue differentiation model of Reina-Romo et al. [24] that was used in this study.

Bone regeneration during DO was simulated with a fuzzy logic-based mechano-regulatory tissue differentiation algorithm in an iterative manner. In the mechano-regulatory tissue differentiation algorithm, the callus area was discretized into finite elements. Each element of the callus was assumed to be a mixture of four tissue types (connective tissue, cartilage, woven bone, and cortical bone). Using the fuzzy logic controller of MATLAB (Fuzzy Logic Toolbox in MATLAB, The MathWorks, Inc., Natick, MA, USA), the process of tissue differentiation is treated as an initial value problem based on two mechanical (dilatational (ε) and distortional (γ) strains in the mechano-regulatory model) and five biological state variables (blood perfusion, cartilage concentration, and bone concentration, as well as blood perfusion and bone concentration in adjacent elements) [30,31]. All seven state variables were used to predict angiogenesis, endochondral ossification, chondrogenesis, cartilage calcification, and tissue disruption in the callus with a linguistic rule-based fuzzy logic. The rules were based on the mechano-regulatory model proposed by Reina Romo et al. [24] (Figure 10), which can take into account the differences between tension- and compression-governed tissue differentiation of DO. According to the rules of tissue differentiation, the fuzzy logic controller judged the input state (seven state variables) of each element in the callus area, and finally output the changes in blood perfusion, cartilage and bone concentration to predict the results of tissue differentiation [30,31]. Following each step of tissue differentiation, the biological state variables of each callus element were updated, and then the material properties of the callus area were updated by using mixture rules according to the current biological state variables [30,31].

### 4.3. Model Implementation and Validation

The simulation started with a pre-processor run of the finite element program, which generated geometry, element mesh, external fixation, load, and boundary conditions. Initial values for the tissue composition, the material properties and the blood perfusion were assigned to each of the finite elements (Figure 10). The tissues in the callus were assumed to be full of connective tissues at the beginning of the distraction period since, according to experimental data, the four days of latency before the distraction period were not enough for regeneration of new bone tissues [24,25]. The initial callus had no blood perfusion (0%). Bone marrow and cortical fragments were rich in blood vessels and had blood perfusion of 100% [20]. Next, the iteration loop started with the finite element analysis to calculate the patterns of local mechanical strains. These strains, together with the current tissue composition and local blood perfusion, were used as input to the fuzzy logic controller. The biological state variables of the adjacent elements were obtained by judging the weight of adjacent regions through a Gaussian kernel function [30], which was used to represent the propagation range of tissue growth. Fuzzy rules described changes in tissue composition and blood perfusion within each finite element. The material properties of the elements were then updated according to the regenerated new tissue composition [30,31] (Figure 10).

To avoid numerical errors associated with highly deformed elements due to stepwise distractions, the callus was remeshed prior to every new distraction step in the distraction phase. Subsequently, all tissue properties were mapped from the previous mesh onto the new mesh, using a sampling and weighting approach proposed by Niemeyer et al. [30]. The residual stress generated in each analysis step was taken as the initial condition of the next analysis step (Figure 10). The simulation was implemented with python scripting in ABAQUS and MATLAB programming. The outputs of the simulation included the changes of interfragmentary movement (IFM), regenerated bone area (mm^2^), and tissue concentrations (bone, cartilage, and blood perfusion) over the healing time. Bone area was computed as all the bone formed in the callus area.

Our model was validated by the experimental results under a standard constant-rate distraction protocol (1 mm/day, twice a day) [4]. Briefly, Claes et al. [4] created a mid-diaphyseal bony defect of 15 mm in the sheep metatarsus. Following 4 days of latency, they performed bone transport at a distraction rate of 1 mm per day, in 2 increments, for 15 days. During the consolidation period, they used a customized external fixator to examine the effect of the stiffness of the axial fixator on the maturation time of the callus, after completion of the distraction process. The initial dynamic fixation group of 0.5 mm (IFM) was selected for our model validation (the boundary condition at this point was used as the boundary condition for subsequent rate-varying distraction protocols). The tissue differentiation algorithm used in the current study was validated by comparing our model-predicted IFMs and the changes in bone concentration after 12 weeks with those obtained from the experiments [4].

### 4.4. Rate-Varying Distraction Protocols

Two distraction rates were used in the study: low-rate (L: 1 mm/day, twice a day) and high-rate (H: 2 mm/day, twice a day). The constant low-rate distraction protocol (1 mm/day, twice a day, for 15 days), as used in the experiment above, served as a control. The distraction rate of 1 mm/day is generally considered as an optimal distraction parameter [10,11,23,25] and has been widely used in clinical practice [8,14]; we sought to explore if any rate-varying protocols could produce a faster, better healing result in comparison. While maintaining the total distracted length of 15 mm unchanged, these two distraction rates (L and H) were combined in different orders, with varied acting periods (rate-varying distractions from high-to-low rates: H1L13 ~ H7L1 or from low-to-high rates: L1H7 ~ L13H1) (Figure 9b). For example, H1L13 indicated a high-rate distraction for 1 day followed by low-rate distractions for 13 days. An IFM of less than 0.05 mm was indicated bony bridging [20]. The healing outcomes of the rate-varying distraction protocols were compared with those of the controls. 

## Data Availability

The data presented in this study are available on request from the corresponding authors.
